# Address

**Published:** 1879-04

**Authors:** W. H. Morgan


### THE DENTAL REGISTER.

VOL. XXXIII.] APRIL, 1879. No 4.

### ADDRESS.

*At the thirty-third annual Commencement of the Ohio College of Dental Surgery, March 6, 1879.*

BY W. H. MORGAN, D. D. S.

I have the honor to be present with you on this interesting occasion by invitation of your faculty, and have been requested by them to make such remarks as shall seem to me most appropriate to the occasion.

It is always a pleasure to assist in launching my young brethren into professional life, but at this time that pleasure is greatly heightened by the knowledge that we are assembled in a building which we can safely call our own. Heretofore we have been groaning under a bondage of debt, since if it be true that the borrower is slave to the lender, it is no less a fact, in a similar sense, that the debtor belongs to the creditor. Now, by the liberality of our professional brethren, chiefly of this state, that yoke has been removed, and we stand here to-day free men. I congratulate you, gentlemen of the faculty and trustees, most heartily upon this pleasing fact. I congratulate you upon the generosity and liberality which produced such a consummation. This building stands now and shall stand, I hope and believe, for our children's children, a monument to your liberality. The city of Cincinnati has long been distinguished and commended for the excellence and completeness of its public schools, which give evidence of the appreciation of knowledge and information by the whole people. Its fame has spread abroad, for the thoroughness and extent of its private institutions of learning, supplementing that more general instruction imparted by the public to all the rising generation. Your grand Fountain, a marvel of æsthetic art, and your unequaled Academy of Music, each of them the princely work of an individual, are proof sufficient of the higher cultivation of that artistic taste, which smoothes the rough corners of life, and add a piquant zest to all its enjoyments. Our building, finished, furnished and *paid for*, adds another to those achievements of which this city may justly be proud.

The purpose for which this building was erected, and the Ohio College of Dental Surgery established, is a result of the demand of the present day for higher education in the science of medicine. It has not been very many years since such duties as were then considered as belonging to the dental art, were shared between the barber and the surgeon. A higher civilization has elevated our profession to its proper station, and the same cultivation which resulted in your Schools, your Fountain and your Academy of Music, has made Dentistry a specialty of medicine, no longer remitted to the ignorant barber, or carelessly tacked, as an addendum of little consequence, to the duties of the general physician. If civilization has introduced new diseases and causes for decay, it has also brought with it the opportunity of acquiring the skill which shall arrest the one and remove the other. As fever breeding swamps of South America produce, they alone furnish, the providential specific for their own miasmata.

That Dentistry has thus become a specialty is not an isolated fact in the progress of the present generation towards a higher education. We can see the same tendency in all departments of technical learning. There was, and within the recollection of many of us who are not old, a time when the learning, classed under what is called the Natural Sciences, could be mastered readily by a studious youth during his college term, and one instructor was considered sufficient for all its branches. Astronomy, Natural Philosophy, including Optics and Acoustics, Chemistry, Organic and Inorganic, and all cognate subjects, even thirty years ago, in our best Colleges, were grouped together, and a single year was sufficient to furnish a student all he needed to know about them. To-day, how great is the change? The intelligent and unceasing inquiries of great minds have so enlarged the sphere of man's knowledge of nature that now scarce a life time is sufficient to master all that has been accumulated in any one of the branches I have named. But not only has the extent of learning been vastly enlarged; its character has changed. The mechanical arts have been elevated to a position of prominence and importance unknown before. When formerly the strong arm was the chief requirement; now the active and well informed brain is most in demand, and one weak hand, moving machinery devised by the brain of genius, can accomplish what multiplied muscle never could effect. So, by no special design beyond that of an overruling Providence, the characteristic of our day has come to be the division of education into special lines and its determination into certain peculiar channels, according to the life work which each young man had laid out for himself. I lay no claim to prophetic knowledge, but it seems plain to me that the day is rapidly approaching when the lines between these specialties will be more and more clearly and sharply drawn. The indications are that the tendencies in our present education are embryonic of the character of the great University of the future, it may be of the near future, which we shall live to see. In this city, perhaps from this very building as a nucleus, shall rise up that institution in which, from the foundation of the learning of your public schools, shall radiate lines of instruction adapted to particular wants, and which shall qualify in an especial manner those who propose to pursue a particular avocation. It is not necessary to further illustrate the idea, but I now call upon the Trustees and Faculty of this College to hold themselves in readiness to move in this direction whenever the advancing wave of culture may require. Those who attempt to resist the pressure will be submerged and lost, while the readiest to respond will reap the highest rewards.

To you, young gentlemen, who have borne the burden and heat of the scholastic day and passed creditable examination, I tender my sincere congratulations. You receive to-day the honors of this College, and will take away with you the certificates of this institution that you have successfully accomplished the course of study prescribed. But these honors and this diploma, I beg you to remember, are accompanied by grave responsibilities. You occupy now the position of the athlete in the Grecian games, who having been carefully trained, presents himself for the contest. You are now about to begin a struggle in which each one of you will surely take the place to which he is entitled. You have here been only taught *what* to study. The actual labor now begins. To attain excellence, and to preserve it will require of you unceasing exertion. I say to *preserve* it, for excellence is a relative term. The man who to-day stands at the very summit of attainments in our profession, if he shall remain stationary, will in a few short years find himself surpassed, and his mountain of learning dwindled by comparison into a molehill. Let your ideal of perfection be as high as the heavens, and strive continually to reach it. You shall never attain to it, or if you do you may be sure it was too low. No one aims too high. There is no excellence without great labor, and the man who thinks he is high enough is certain to fall.

And while I thus exhort you to keep abreast of the foremust, and devote yourself without ceasing to the attainment of the highest ends of our special profession, I wish also to call your attention to some necessities and duties more general in this character and of personal application.

I have before intimated that the elevation of the specialty of Dentistry to the dignity of a Profession was the result of a higher civilization. On the same principle and for a similar reason you will find that the great mass of those who will apply for your professional services are persons of culture and refinement. As a general rule you will be associated with the educated and accomplished of the communities in which you reside, and it therefore behooves you to be prepared to occupy among them a position commanding respect, aside from your professional knowledge. To this end, cultivate the study of sound and healthy literature. Read good books; and there are plenty of them, so many that he who reads trash is inexcusable. Never take up the foolish, trashy novel nor the vapid, sensational magazine, but read the works of standard authors, history, fiction, general literature, and be prepared to feel and to make it known in the company of your educated and refined patients, that you also are refined and educated. The pursuit of your professional studies, which must for no cause be interrupted, and the cultivation and improvement of your literary knowledge and taste, will leave you no idle moments, and while they will insure your professional success, they will, at the same time, render certain your personal happiness. They will do this both affirmatively and negatively, by furnishing constant occupation and thus shutting out the many temptations which attack the idle and the aimless.

There is one equality with which some are more largely endowed by nature than others, which the educated Dentist should sedulously cultivate, and that is *sympathy*. There is no profession whose practitioners are required so generally to inflict pain upon those they propose to benefit. It is a commonly received opinion that no painless dental operation can be performed without the use of an anæsthetic. Sympathize, not only in words, but really and heartily with all who are thus compelled to suffer, and especially with the delicate, nervous and morbidly sensitive. Soothe by mildness and honest compassion; never by ridicule or slighting words or acts, or any other form of harshness, attempt to drive a patient. But, nevertheless, perform your duty. When pain must be inflicted, or your work imperfectly done, without hesitation choose the former. In short, the educated and skillful Dentist should have the tender heart and hand of a woman, and the unyielding firmness and steady nerve of the stalwart man.

Having received the honors of the profession, and being about to enter upon the active duties of life in the practice of it, you are in honor bound to maintain its dignity and advance its interests. This you can best do by supporting its institutions of learning, by attending and participating in its associations and by adhering most strictly to the code of ethics established among its members. By supporting its institutions of learning you insure their stability and prosperity and thus afford the means to all who desire to enter our ranks to properly qualify themselves to do so, and thus prevent indirectly the increase of quacks and charlatans. At its associations are collected together the best minds, the greatest learning, the highest skill in the profession, and as one of the most striking characteristics of true greatness and knowledge is the generosity with which all enquirers after the truth are met, you will find that, next to the value of the lectures by the professors of your college, will be the essays and discussions in your associations.

The ethics of the profession are founded upon the Golden Rule—Do unto others as you would have them do unto you. The regulations constituting the code have not been made by a single man. They are like the common law, the product of the combined wisdom of very many men laboring through many years to fix a correct standard, and as a whole generation is wiser than one man, these rules are the more likely to be always right, and I enjoin upon you the strict observance of every one of them.

While you thus devote yourselves to the advancement of your profession and to the attainments of the rewards it offers, keep your personal character pure and spotless, and every appearance of vices, as they are termed, as you would the minutest particle of a contagion.

I desire especially to impress upon you that the purest code of morals, the safest rules for personal conduct and the best system of sanitary regulations known to man are given us in the Word of God. Read it, study it, however pressing and onerous your engagements may be, keep it always by your side, follow its precepts, and whether the measure of your success be small or large, you will be happy in its enjoyment.

In conclusion, gentlemen, I greet you as professional brethren, I extend to you the right hand of fellowship. Armed with the learning of your preceptors, fortified with the principles of professional and personal honor and virtue, qualified for and equal to the society of the refined and educated amongst whom your practice will be, I commend you to the regard of the brethren everywhere and the guidance of Providence. May you be succesful as practitioners, honored as men, and ornaments to the profession.

